# Partially Separable Aspects of Spatial and Temporal Estimations in Virtual Navigation as Revealed by Adaptation

**DOI:** 10.1177/20416695221078878

**Published:** 2022-02-24

**Authors:** Taku Otsuka, Yuko Yotsumoto

**Affiliations:** Department of Life Sciences, 13143 The University of Tokyo, Tokyo, Japan; Department of Life Sciences, 13143 The University of Tokyo, Tokyo, Japan

**Keywords:** time perception, distance perception, distance reproduction, magnitude estimation, adaptation

## Abstract

Recent studies claim that estimating the magnitude of the spatial and temporal aspects of one's self-motion shows similar characteristics, suggesting shared processing mechanisms between these two dimensions. While the estimation of other magnitude dimensions, such as size, number, and duration, exhibits negative aftereffects after prolonged exposure to the stimulus, it remains to be elucidated whether this could occur similarly in the estimation of the distance travelled and time elapsed during one's self-motion. We sought to fill this gap by examining the effects of adaptation on distance and time estimation using a virtual navigation task. We found that a negative aftereffect occurred in the distance reproduction task after repeated exposure to self-motion with a fixed travel distance. No such aftereffect occurred in the time reproduction task after repeated exposure to self-motion with a fixed elapsed time. Further, the aftereffect in distance reproduction occurred only when the distance of the adapting stimulus was fixed, suggesting that it did not reflect adaptation to time, which varied with distance. The estimation of spatial and temporal aspects of self-motion is thus processed by partially separable mechanisms, with the distance estimation being similar to the estimation of other magnitude dimensions.

In the real world, spatial and temporal information is often correlated. For example, when navigating in an environment, it naturally takes a long time to travel a long distance. Therefore, their perceptual mechanisms may be closely related. According to A Theory Of Magnitude (ATOM), space and time, together with other magnitudes such as numerosity, are processed by a generalized magnitude system, primarily in the parietal cortex (Bueti & Walsh, 2009; Walsh, 2003). Evidence supporting this hypothesis comes from multiple behavioural studies examining the interference effect between the perception of space and time. Their findings suggest that humans are unable to ignore irrelevant spatial information when estimating time (Brunec et al., 2017; Brunec et al., 2020; Casasanto et al., 2010; Casasanto & Boroditsky, 2008) and cannot ignore irrelevant temporal information when estimating space (Cai & Connell, 2015; Cai & Wang, 2021; Riemer et al., 2018). In addition to behavioural findings, multiple neuroimaging studies have suggested that a common neural mechanism is responsible for spatial and temporal processing. For example, fMRI studies have consistently revealed that overlapping regions of the parietal cortex are activated in these two domains (Coull & Nobre, 1998; Dormal et al., 2012; Peer et al., 2015; Skagerlund et al., 2016).

However, the extent to which the abovementioned domains overlap, as well as their specific mechanisms, remains to be clarified. For example, it has been shown that the interference effect between spatial and temporal perceptions is often asymmetric; in many cases, the perception of time is influenced by spatial information, while the perception of space is less or not affected by temporal information (Casasanto et al., 2010; Casasanto & Boroditsky, 2008; Riemer et al., 2018). These findings suggest that, while these two dimensions are indeed related to each other, their processing mechanisms are to some extent separable. A recent meta-analysis of neuroimaging studies supports this view. Cona et al. (2021) suggested that brain regions for space and time processing partially overlap, and that each neural representation forms a gradient across the cortex.

Among spatial and temporal perception, accurately estimating the distance and time of one's self-motion is crucial for navigation (Glasauer et al., 2007). Recently, based on the above findings on the processing of space and time as a magnitude estimation, the characteristics of the estimation of distance and time, and their neural substrates, have been studied using the virtual reality (VR) environment in humans and rodents (Kautzky & Thurley, 2016; Petzschner & Glasauer, 2011; Riemer et al., 2018; Robinson et al., 2019; Robinson & Wiener, 2021; Thurley & Schild, 2018; Wiener et al., 2016). In the virtual distance reproduction task, participants moved a specific distance or time in the VR environment and then reproduced the distance or time (Petzschner & Glasauer, 2011; Robinson & Wiener, 2021; Thurley & Schild, 2018; Wiener et al., 2016). Using this paradigm, it has been reported that both reproduced distance and reproduced time are subject to similar characteristic biases, such as the central tendency and range effects (Petzschner & Glasauer, 2011; Robinson & Wiener, 2021; Thurley & Schild, 2018). This may indicate that the estimation of distance and time are explained in a unified Bayesian framework for magnitude estimation (Petzschner et al., 2015) and that similar processing mechanisms are recruited in these two dimensions, as suggested by ATOM (Bueti & Walsh, 2009; Walsh, 2003).

However, while similar characteristics are known for distance and time estimation, whether they are processed by shared or separate systems is still under debate. Accumulating evidence has shown that there is an asymmetric or independent relationship between the estimation of travel time and travel distance (Brunec et al., 2017; Riemer et al., 2018; Robinson et al., 2019) and between their neural substrates (Liang et al., 2020; Robinson & Wiener, 2021). At the lower levels, perception of travel time is influenced by distance, whereas perception of travel distance is not affected by time, which is consistent with the view that perception of temporal features is susceptible to spatial features, but not vice versa (Riemer et al., 2018). Furthermore, at higher levels of decision making, the distance and time to reach the reward are estimated independently in reward discounting (Robinson et al., 2019). In addition, EEG studies have indicated that distance and time are processed by distinct coding schemes in terms of ERPs (Robinson & Wiener, 2021) and oscillations (Liang et al., 2020). These findings suggest that travel distance and travel time are closely related, but may be processed by partially separable systems.

In the estimation of different magnitudes, previously perceived stimuli can cause characteristic biases in the estimation of the current stimulus. One of them is the central tendency effect described above, in which the estimation of the current stimulus regresses to the mean of the distribution of stimuli. Other examples are the serial dependence and adaptation effects. In particular, the adaptation effect, more specifically the negative aftereffect, is a phenomenon in which prolonged exposure to certain stimuli biases the estimation of the current stimulus in the direction that is repulsive to the preceding stimulus; after prolonged exposure to a stimulus of relatively small magnitude, a subsequent stimulus of relatively large magnitude is judged to be even larger, and vice versa. Negative aftereffects are known to occur in various magnitudes, such as size (Pooresmaeili et al., 2013), numerosity (Burr & Ross, 2008), and duration (Heron et al., 2012; Shima et al., 2016). Although channel-based mechanisms that selectively encode a particular property have been suggested as the neural basis for this phenomenon (Castaldi et al., 2016; Hayashi & Ivry, 2020; Heron et al., 2012; Pooresmaeili et al., 2013), they may originate from higher-level cognitive effects related to memory and decision biases, such as the anchor effect (Postman & Miller, 1945; Storrs, 2015). However, to date, whether negative aftereffects can occur in estimating the spatial and temporal aspects of one's self-motion has not been investigated. Regardless of the specific mechanism, given the similarity between the estimation of travel distance and travel time and other magnitudes, we can predict that a negative aftereffect may also occur in the distance and time estimation. In addition, if the mechanisms of distance and time estimation are shared, we would predict that their adaptation effects show similar characteristics.

The purpose of this study was to investigate the similarities and differences in the estimation mechanisms of travel distance and travel time by examining adaptation effects. We hypothesized that negative aftereffects would occur in the estimation of travel distance and travel time, and that their adaptation effects would show similar characteristics. To test this hypothesis, using a virtual distance/time reproduction task, we investigated how the estimation of travel distance or travel time is affected by repeated exposure to self-motion with fixed distance or fixed time. For this purpose, we attempted to separate the distance adaptation (i.e., fixed distance, variable time) from time adaptation (i.e., fixed time, variable distance) by randomly varying the moving speed. In Experiment 1, we examined whether time adaptation produces a negative aftereffect in estimating time and whether distance adaptation produces a negative aftereffect in estimating distance. In Experiment 2, we further examined whether the aftereffect for distance estimation, which we observed in Experiment 1, could result from the adaptation to time that covaried with distance.

Reported data and analysis scripts from all experiments are available on the Open Science Framework (https://osf.io/p2ubr/).

## Experiment 1

The aim of this experiment was to examine if a negative aftereffect occurs when estimating the distance travelled and time elapsed for one's self-motion.

### Method

#### Participants

An a priori power analysis was conducted using G*Power 3.1 (Faul et al., 2007) to detect within-subject effects given repeated measures. We used five as the number of conditions per participant, a medium effect size (f  =  0.25), an alpha of 0.05, and default values for correlations among repeated measures and non-sphericity corrections. A total sample size of 20 was required to achieve a power of 0.80. Therefore, we recruited twenty participants in Experiment 1 (twelve males; nineteen right-handed; mean age  =  20.1 years, SD  =  1.51). All participants had normal or corrected-to-normal vision. We excluded one participant (see Data analysis), and the data of the remaining 19 participants were used for the analysis. All participants voluntarily participated in the experiment with 1,000 Japanese yen (JPY) per hour as payment and provided written informed consent before the experiment. The protocol was approved by the Institutional Review Board of the University of Tokyo.

#### Apparatus

The stimuli were coded in C# using Unity version 2018.4.27 (Unity Technologies, Inc.) and The Unity Experiment Framework (Brookes et al., 2019) and presented on a 23.6 inch LCD monitor with 1920  ×  1080 resolution at a refresh rate of 120 Hz (VIEWPixx 3D; VPixx Technologies, Inc.). Stimulus presentation and data recording were controlled using a workstation (Windows 10 Professional 64bit). Participants were seated in a chair and their heads were fixed on a chin rest 57.3 cm away from the monitor. A keyboard for recording the participants’ responses was placed between the monitor and the participants. The experiments were conducted in a dark room.

#### Stimuli

**Figure 1.**
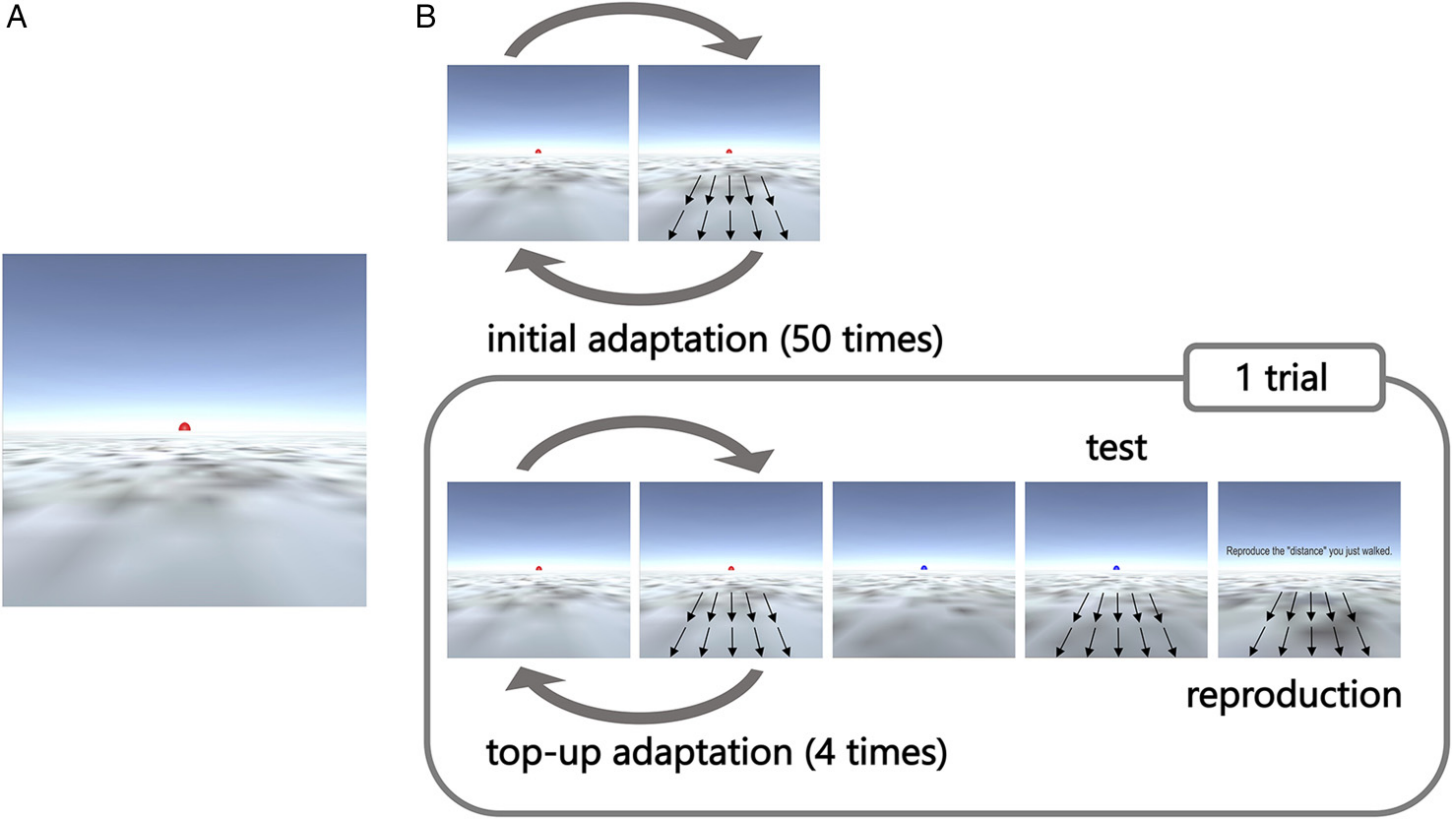
Experimental design. A: Example of the stimuli used for the time and distance reproduction task. The virtual reality environment consisted of ground and blue sky with no sun or clouds, and no spatial cues. B: Experimental procedures used in Experiments 1 and 2. The stimuli depicted in the figure represent an example of the distance reproduction task.

The VR environment and the experimental task were modelled after previous studies (Petzschner & Glasauer, 2011; Robinson et al., 2019; Robinson & Wiener, 2021; Thurley & Schild, 2018; Wiener et al., 2016). The VR environment was designed to mimic a desert environment and consisted of ground (52  ×  14.5°) and sky (52  ×  14.5°). The ground texture was generated using Unity assets (World Materials Free) to avoid repeating patterns, and the sky was presented as a blue sky with no sun or clouds. Objects such as rocks and landmarks that could provide distance clues were not placed. The starting position of each movement was randomized in each trial, and the eye height of the VR viewpoint was adjusted to approximately match the eye height of the participant. A red hemisphere with a 1.3°diameter was placed in the centre of the screen for fixation (Figure 1).

#### Procedure

Experiment 1 consisted of distance adaptation–distance reproduction task (DADR) and time adaptation–time reproduction task (TATR). DADR and TATR were each tested in one session, and the order was counterbalanced across participants.

#### Distance Adaptation–Distance Reproduction task (DADR)

In DADR, the travel distance of the test stimulus (test distance) was fixed at 2.4 m in metres in Unity, and the travel distance of the adapting stimulus (adapting distance) was varied in five steps. To examine the aftereffects in both directions, two adapting distances were shorter than the test distance, two were longer than the test distance, and one was equal to the test distance (baseline condition). The resulting five adapting distances were 1.0, 1.5, 2.4, 3.8, and 6.0 m. Note that the travel distances were determined arbitrarily based on a metric of the Unity software used to create the VR environment. Therefore, the perceived distance did not necessarily match the one in the real world.

The experiment consisted of one session and five blocks. Each block consisted of initial adaptation and test trials. The adapting distance within a block was held constant. The number of test trials in each block was 60, and as a result, all participants performed 60 test trials for each adapting distance. The order of the blocks was randomized within and across participants.

The experimental procedure is illustrated in Figure 1. At the beginning of each block, participants observed the adapting stimulus 50 times as an initial adaptation. The adapting stimulus was a forward movement that simulated walking. The viewpoint moved in the direction of the red sphere presented on the horizon of the VR environment and stopped when it reached the adapting distance. The test trials followed initial adaptation. One test trial consisted of four top-up adaptations, a test stimulus presentation, and a reproduction of the test distance. Before the test stimulus was presented, the colour of the sphere changed from red to blue as a cue. After the test stimulus was presented, the instruction “Reproduce the distance you just walked” appeared at the centre of the screen, and participants were allowed to reproduce the test distance by pressing a button with their dominant index finger until they had reproduced the same distance as the test distance. The inter-stimulus interval (ISI) between the initial adaptation and the first test trial was 2000ms, and the other ISIs (between the adapting stimulus, between the adapting and the test stimulus, and between the test stimulus and the instruction for distance reproduction) were randomly jittered in the range of 500 to 1000 ms.

Importantly, for all movements, the moving speed was randomly drawn from a uniform distribution [2.5 m/s, 4.5 m/s]. By doing so, we attempted to minimize the adaptation to the travel time, namely, the travel distance of the adapting stimulus was fixed, while the travel time was varied within the block. In addition, we attempted to prevent participants from reproducing the test distance based on the travel time of the stimulus. During the task, participants were instructed to gaze at the red sphere in the centre of the screen, but were allowed to move their gaze to some extent in order to grasp the distance information. They were also instructed not to count the duration of the test stimulus (Rattat & Droit-Volet, 2012) and not to memorize or reproduce the test distance based on the duration of the stimulus or the texture of the ground. The participants were informed that the texture of the ground and the moving speed would change during every movement.

Before the session, participants underwent a practice block to familiarize themselves with the reproduction task procedure. The practice block was the same as the actual experiment, except that it consisted of only three times the initial adaptation for each adapting stimulus, two top-up adaptations, and 12 test trials in each block.

#### Time Adaptation–Time Reproduction task (TATR)

The procedure for TATR was similar to that of DADR, except that the movement of the adapting stimulus stopped when it reached the adapting time, and the participants reproduced the test time, rather than distance after the instruction “Reproduce the time you just walked” appeared on the screen, following the presentation of the test stimulus.

The travel time of the test stimulus (test time) was fixed at 0.7 s, and the travel time of the adapting stimulus (adapting time) was varied in five steps. To make the results comparable with those of DADR, the five adapting times were set to 0.3, 0.45, 0.7, 1.1, and 1.7 s. These values were determined so that the adapting time would be close to the value of the adapting distance in DADR divided by 3.5 m/s, which is the expected value of the distribution of the moving speed.

As in DADR, the moving speed was randomly drawn from a uniform distribution [2.5 m/s, 4.5 m/s]. In this case, we attempted to minimize the adaptation to travel distance. In addition, we attempted to prevent participants from reproducing the test time based on the travel distance of the stimulus. They were instructed not to count the duration of the test stimulus (Rattat & Droit-Volet, 2012) and not to remember or reproduce the test time based on the distance travelled or the texture of the ground.

#### Data analysis

For data exclusion, we used the same criteria for the DADR and TATR. For each participant, trials in which reaction time was greater than the median  ±  3 SDs (Miller, 1991), trials in which the reaction time was less than 100 ms, and trials in which the reproduced duration was greater than the median  ±  3 SDs were excluded from the analysis.

In DADR/TATR, if the adapting distance/time is equal to the test distance/time, no aftereffect is expected to occur. Thus, we treated this condition as the baseline condition. For each participant, we calculated the mean of the reproduced distance/time for the baseline condition and divided the reproduced distance/time for the other conditions by this value (Shima et al., 2016). We used this *normalized reproduced distance/time* as the dependent variable. If the normalized reproduced distance/time is higher than 1, it indicates an overestimation of the test distance/time relative to the baseline; if it is lower than 1, it indicates an underestimation. The normalized reproduced distance/time directly quantifies the magnitude of the adaptation effect and can be compared across different dimensions as it is unitless.

Before the main statistical analyses, we examined the influence of the task-irrelevant dimension on the responses. Specifically, we calculated nonparametric Spearman correlations, for each participant and each adapting condition in each task, between the change in moving speed from the test phases to the reproduction phase and the reproduced distance/time (Hayashi et al., 2013; Robinson & Wiener, 2021; Wiener et al., 2016). This is because, for example, in distance reproduction, although the moving speed was randomly varied between the test phase and the reproduction phase, some participants may have reproduced the distance by matching the duration of the test stimulus (Mossio et al., 2008; Wiener et al., 2016). Similarly, in time reproduction, participants may have reproduced time based on distance information (Riemer et al., 2018). In previous studies, the speeds in the test and reproduction phases were largely different, and it was unlikely that the participants used the task-irrelevant information as a cue (Petzschner & Glasauer, 2011; Robinson & Wiener, 2021; Thurley & Schild, 2018; Wiener et al., 2016). However, in the present experiment, these strategies were partially possible because the speed was drawn from the same uniform distribution in both the test and reproduction phases. Therefore, evaluations of the task-irrelevant dimension are necessary. In the time reproduction task, the correlation would be negative if the response was influenced by the distance of the test stimulus. On the other hand, if the participants reproduced the distance based on the duration of the test stimulus, the correlation would be positive in the distance reproduction task. In addition, to test whether the distance and time tasks matched for difficulty, we calculated the coefficient of variation (CV) for each adapting condition in each task, by dividing the standard deviation of the reproduced distance/time by the average reproduced distance/time.

To evaluate the effect of different adapting conditions on the normalized reproduction distance/time, we created linear mixed-effects models (LMMs) in R version 3.6.2 (R Core Team, 2019) using the lme4 (Bates et al., 2015), lmerTest (Kuznetsova et al., 2017), and emmeans (Lenth, 2021) packages. Compared with traditional repeated-measures ANOVAs in which comparisons are made among averaged data, LMMs are more powerful, as they take single-trial observations into account, and variance between participants is considered as a random effect. Another advantage of using LMMs in this study is that they can incorporate categorical and quantitative variables into the model simultaneously.

We first analyzed the results separately for the DADR and TATR. We entered the normalized reproduction distance/time for each trial as dependent variable, the adapting distance/time as fixed-effect factor, and participant as random-effect factor. In addition, speed change (mean-centreed) for each trial was entered as covariate, as suggested by the correlation analyses (Supplementary Figure 1 and 2), that change in speed also affects the reproduced distance/time. We also included an interaction term to examine whether the effect of speed change was different for each adapting condition. Next, to further investigate whether distance adaptation and time adaptation had different effects on the normalized reproduced distance/time, we created additional LMMs with the normalized reproduced distance/time as dependent variable, and with task (2 levels: DADR or TATR), adapting condition (5 levels), and their interaction term as fixed-effect factors.

**Figure 2.**
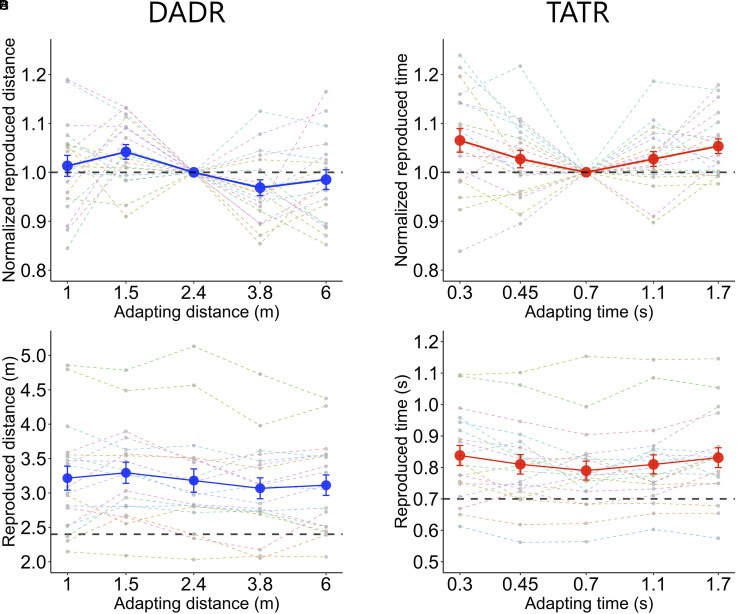
The results of experiment 1. A, B: The average normalized reproduced distance and average normalized reproduced time for DADR and TATR, respectively. The centre disk in each figure indicates the normalized reproduced distance/time after adapting to the same distance/time as the test distance/time (baseline). If the normalized reproduced distance is greater than 1, it indicates that the test distance is overestimated relative to the baseline; if it is less than 1, it indicates that the test distance is underestimated relative to the baseline. C, D: The average reproduced distance and average reproduced time for DADR and TATR, respectively. The black dotted line corresponds to the test distance/time. Gray dots indicate the data for each participant. Error bars indicate SE. DADR  =  distance adaptation–distance reproduction. TATR  =  time adaptation–time reproduction.

All LMM analyses were performed according to the following steps: First, we created a simple random-effects model in which only the mean of the dependent variable was assumed to be different for each participant (i.e., random intercept model), and only the dependent variable and the intercept were contained. From this simple model, the predictors were added incrementally to determine whether the predictor improved the model. Model selection was based on the likelihood ratio test, and the statistical significance of the fixed-effect parameters was tested with F-tests from the selected best models, using lmerTest package (Kuznetsova et al., 2017). Post-hoc tests were performed with t-tests on estimated marginal means (Searle et al., 1980) estimated from the best models and corrected for multiple comparisons using Tukey's HSD, using the emmeans package (Lenth, 2021). The Degrees-of-freedom for F-tests and t-tests were calculated by Satterthwaite's method. The full scripts for the LMM analyses are available on the Open Science Framework (https://osf.io/p2ubr/).

It should be noted that these random intercept models have a high probability of type 1 error. Hence, to avoid reporting spurious fixed effects, it is essential to examine the fixed effects in the presence of the corresponding random slopes (Bates et al., 2018; Matuschek et al., 2017). Therefore, we also created more complex random-effects models, including random slopes for those fixed-effect factors that were significant. Whenever the more complex models were favoured over the simpler models, we reported the results of the complex random-effects models.

In addition, we examined the across-participant correlations of the adaptation effects for distance and time (Anobile et al., 2018): Separately for each of the four adapting conditions, we obtained the mean of the normalized reproduced distance/time and calculated the Pearson correlation coefficient. The significance of correlations was calculated by p-value and also by Bayes Factor (Wetzels & Wagenmakers, 2012) using JASP (JASP Team, 2020).

### Results

As a result of our data exclusion procedure, we excluded one participant from further analysis, whose data of more than 40% of the trials were excluded for both DADR and TATR. Of the remaining data for 19 participants, 3.6% and 3.9% of the trials were excluded from the analysis for DADR and TATR, respectively.

#### Effects of task-irrelevant dimension on distance and time reproduction

Correlation analysis examining the relationship between speed change and reproduction revealed that task-irrelevant dimensions (distance or time) affected the primary task responses in DADR and TATR (Supplementary Figure 1). In DADR, the across-conditions mean of the correlations between speed change and reproduced distance was significantly different from 0 (mean Spearman's rho  =  -0.18, SD  =  0.29, t(18)  −2.67, p  =  .015), suggesting a time-on-distance effect. In TATR, the across-conditions mean of the correlations between speed change and reproduced time was also significantly different from 0 (Spearman's rho  =  -0.46, SD  =  0.17, t(18)  −11.71, p < .001), suggesting a distance-on-time effect. A comparison of these two effects showed that the distance-on-time effect was greater than the time-on-distance effect (t(18)  4.48, p < .001). Based on these results, we included speed change as a covariate in the following LMM analyses. Additionally, the across-conditions mean of the CV was higher for the distance task than for the time task (t(18)  5.78, p < .001, Cohen's *d*  =  1.18), suggesting that the distance reproduction was less precise than the time reproduction.

#### Distance and time adaptation effects

Figure 2 shows the results of Experiment 1. Figure 2A and 2B show the average normalized reproduced distance for DADR and the average normalized reproduced time for TATR, respectively. In Figure 2A and 2B, for each participant and condition, we divided the reproduced distance/time by the mean of the reproduced distance/time for the baseline condition, where the adapting distance/time was equal to the test value. This normalized reproduced distance/time directly indicates the magnitude of the over/underestimation relative to the baseline, i.e., the magnitude of the adaptation effect. Figure 2C and 2D show the actual reproduced distance/time. The participants tended to overestimate the test value in both the distance and time tasks. However, it should be noted that, since each participant has a different baseline for over/underestimation of the test value, the actual reproduced values shown in Figure 2C and 2D do not reflect the effect of the adapting stimuli. The effect of adapting distance on the normalized reproduced distance was significant for DADR (F(4, 20.1)  7.83, p < .001), and the effect of adapting time on the normalized reproduced time was significant for TATR (F(4, 19.6)  10.92, p < .001). The interaction between speed change and the adapting condition was not significant for either DADR or TATR, suggesting that the difference between conditions cannot be explained by the effect of task-irrelevant dimensions on the responses. If adaptation causes a negative aftereffect on the estimation, then the reproduced distance and time would be longer after adapting to shorter magnitude and shorter after adapting to longer magnitude, resulting in a negative linear (Shima et al., 2016) or a cubic trend (Becker & Rasmussen, 2007). In line with this prediction, post-hoc polynomial contrast tests confirmed that for DADR, the data had significant linear (t(19.0)  −4.32, p < .001) and cubic trends (t(18.9)  2.37, p  =  .028). Post-hoc pairwise comparisons indicated that the normalized reproduced distance was greater when the adapting distance was 1.5 m compared with when the adapting distance was 3.8 m or 6 m (t(19.0)  3.59, p  =  .014; t(19.0)  3.53, p  =  .016, respectively). In contrast, for TATR, post-hoc polynomial contrast tests showed that the data had a significant quadratic trend (t(21.1)  6.20, p < .001); however, the linear and cubic trends were not significant (t(18.9)  −0.29, p  =  .768; t(19.1)  0.32, p  =  .751, respectively). Post-hoc pairwise comparisons indicated that the normalized reproduced time was greater when the adapting time was 1.7 s compared with when the adapting time was 0.7 s (t(19.4)  3.440, p  =  .020).

These results suggest that the estimation of travel distance and travel time show different estimation biases after repeated exposure to self-motion. Consistent with this interpretation, further LMM analyses showed that the interaction between task (two levels: DADR or TATR) and adapting condition (five levels) was significant (F(4, 21.7)  5.97, p  =  .002). However, an additional correlation analysis (Anobile et al., 2018) did not provide conclusive evidence to support either a correlation or a lack of correlation between the adaptation effects for distance and time (detailed descriptions are available in the supplementary material).

### Discussion

Correlation analysis showed that the task-irrelevant dimension of the stimulus affected the primary task response in both the distance and time reproduction tasks, but these effects were asymmetric. In other words, the effect of distance on time was larger than the effect of time on distance. This is consistent with a study showing that time perception is sensitive to distance, but distance perception is resistant to time in virtual navigation (Riemer et al., 2018). In contrast, other previous studies using similar task designs have reported almost no effect or that the time-on-distance effect is slightly larger than the distance-on-time effect (Kautzky & Thurley, 2016; Robinson & Wiener, 2021; Thurley & Schild, 2018; Wiener et al., 2016). This difference might be due to the range of speeds used or the presence of feedback. Unlike in the current experiment, in the previous studies, the speed change between the test stimulus and the reproduction phase was noticeably large, and/or feedback was given after the reproduction to prevent participants from reproducing distance/time based on duration/distance. However, because the effect of task-irrelevant dimensions is considered in the LMM analyses, we believe that they do not invalidate the main results. On the other hand, the CV of the distance task was higher than that of the time task, suggesting that the distance reproduction was less precise than the time reproduction. Since we did not match the difficulty of the tasks explicitly, this difference may have led to the differences in the adaptation effects between the spatial and temporal estimation.

In DADR, the reproduced distance followed a significant linear and cubic trend, with a tendency for longer reproduced distance when the adapting distance was shorter than the test distance, and shorter reproduced distance when the adapting distance was longer than the test distance. These are consistent with the characteristics of typical negative aftereffects (Becker & Rasmussen, 2007; Shima et al., 2016), thus supporting the hypothesis that negative aftereffects occur in the estimation of travel distance.

In TATR, contrary to our predictions, there was a significant quadratic trend; overestimation was observed in the conditions where the adapting time was shorter than the test time and where the adapting time was longer than the test time. If a negative aftereffect occurs, underestimation should be observed in conditions where the adapting time is longer than the test time, as in DADR. Therefore, this result does not support the hypothesis that an aftereffect occurs in the estimation of travel time. While this finding seems inconsistent with previous studies that showed negative aftereffects for duration perception (Heron et al., 2012; Li et al., 2017; Maarseveen et al., 2019; Shima et al., 2016), we would like to emphasize that simple comparison is not possible, because previous studies used simple non-contextual stimuli, whereas the present study used context-dependent stimuli that were accompanied by distance and speed information. A possible explanation for the observed quadratic trend remains speculative, but will be discussed in the general discussion.

The results of Experiment 1 suggest that aftereffects similar to those of other reported magnitude estimations occur when estimating travel distance, but not when estimating travel time. The fact that different estimation biases were observed for the distance and time reproductions suggests that travel distance and travel time are estimated through distinct processing mechanisms. If the perceptual mechanisms for space and time are independent, the magnitude of the adaptation effects would not be correlated (Anobile et al., 2018). However, the additional correlation analysis did not provide conclusive evidence to support either a correlation or a lack of correlation. Thus, we are not able to argue the independence of the mechanisms for space and time from these correlations.

One may think that the observed distance aftereffect might simply reflect adaptation to the speed of movement. However, as the range of speeds used in all the adapting conditions was drawn from the same uniform distribution, the observed effects cannot be explained by speed adaptation. The absence of an interaction between speed change and adapting condition suggests that the differences in results between conditions cannot be explained by the effect of the task-irrelevant dimension, either in DADR or TATR. However, it is unclear whether the aftereffect is indeed the result of adaptation to distance. This is because, although we tried to separate adaptation to distance from adaptation to time by varying the moving speed within conditions, adapting distances are correlated with travel time across conditions due to the speed manipulation described above. In other words, the observed aftereffect may have reflected the effects of adaptation to travel distance, travel time, or a combination of these two. Therefore, in Experiment 2, we examined whether adaptation to distance and adaptation to time could be dissociated.

## Experiment 2

The results of Experiment 1 suggest that a negative aftereffect occurs for the distance estimation. However, because the distance and time of the adapting stimuli were correlated across conditions, we could not infer whether it was a result of adaptation to distance or time. To examine whether the distance aftereffect was indeed induced by adaptation to distance, we conducted a cross-dimension adaptation experiment. Specifically, we examined the biases in the distance reproduction when the distance of the adapting stimulus was fixed (DADR, as in Experiment1) and when the time of the adapting stimulus was fixed (time adaptation–distance reproduction: TADR). We hypothesized that, if the distance aftereffect is the result of selective adaptation to the travel distance rather than travel time, the aftereffect would be smaller or not present in TADR.

### Method

Participants. Similar to Experiment 1, an a priori power analysis was conducted using G*Power 3.1 (Faul et al., 2007) to determine the necessary sample size to replicate the results of DADR in Experiment 1. For this purpose, we calculated the effect size f of the factor adapting distance based on Cohen (1992). We used this calculated effect size (f  =  0.48), an alpha of 0.05, and the default values for the correlation between repeated measures and non-sphericity correction for the calculation. When the number of repeated measures was 3, the results showed that a total sample size of nine was required to achieve a power of 0.80. Thus, we recruited ten new participants in Experiment 2 (six males; all were right-handed; mean age  =  21.9 years, SD  =  1.73). All participants had normal or corrected-to-normal vision. They voluntarily participated in the experiment with 1,000 Japanese yen (JPY) per hour as payment and were provided written informed consent before the experiment. The protocol was approved by the Institutional Review Board of the University of Tokyo.

#### Apparatus, stimuli, and data analysis

The same apparatus, stimuli, and data analysis procedures as those used in Experiment 1 were used.

#### Procedure

Experiment 2 consisted of a distance adaptation–distance reproduction task (DADR) and time adaptation–distance reproduction task (TADR). DADR and TADR were each tested in one session, and the order was counterbalanced across participants. The procedure was the same as that in Experiment 1, except for the following. To reduce the experimental time, only three adapting conditions were tested for both the DADR and TADR. In addition, to make DADR and TADR comparable, the adapting distances and times were set to correspond exactly to each other given the expected value of the moving speed (3.5 m/s). This means that within the corresponding conditions, the adapting stimuli move the same distance and time on average, but the time varies in DADR and the distance varies in TADR. For DADR, the adapting distances were 1.54, 2.45, and 3.85 m. For TADR, the adapting times were 0.44, 0.7, and 1.1 s. The test distance was 2.45 m for both DADR and TADR.

### Results

Using the same data exclusion criteria as in Experiment 1, 2.9% and 3.3% of the trials were excluded from the analysis for DADR and TADR, respectively.

#### Effects of task-irrelevant dimension on distance reproduction

The across-condition mean of the correlations between speed change and reproduced distance was not significant in either DADR (mean Spearman's rho  =  -0.05, SD  =  0.31, t(9)  −0.52, p  =  .613) and TADR (mean Spearman's rho  =  -0.07, SD  =  0.36, t(9)  −0.69, p  =  .502), and the effects in the two conditions were not significantly different (t(9)  0.43, p  =  .673) (Supplementary Figure 2) . Although the effect was not significant, speed change was incorporated as a covariate in the subsequent LMM analysis, as in Experiment 1. The across-conditions mean of the CVs for DADR and TADR was not significantly different (t(9)  −0.38, p  =  .708, Cohen's *d*  =  0.11).

#### Effects of distance and time adaptation on distance reproduction

**Figure 3.**
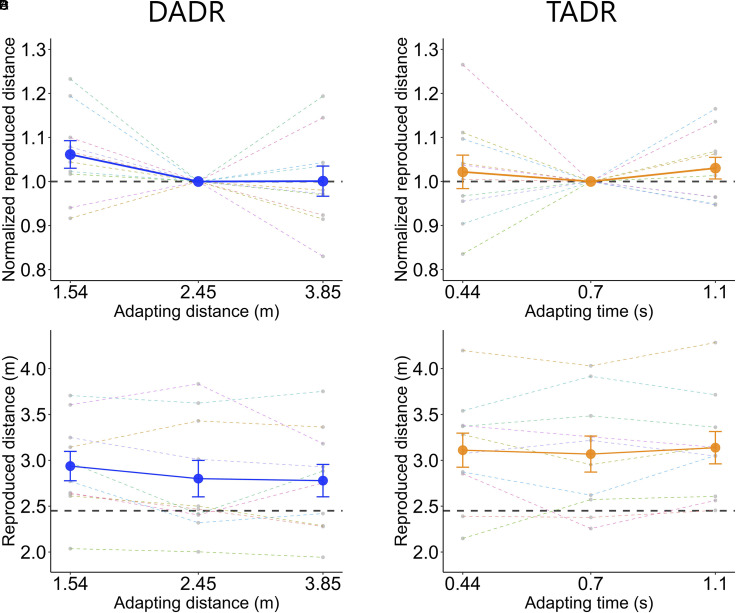
The results of experiment 2. A, B: The average normalized reproduced distance for DADR and TADR. The centre disk in each figure indicates the normalized reproduced distance for the baseline condition. C, D: The average reproduced distance for DADR and TADR. The black dotted line corresponds to the test distance. Gray dots indicate the data for each participant. Error bars indicate SE. DADR  =  distance adaptation–distance reproduction. TADR  =  time adaptation–distance reproduction.

Figure 3 shows the results of Experiment 2. Figure 3A and 3B show the average normalized reproduced distance for DADR and TADR, respectively. Figure 3C and 3D show the reproduced distance without normalization. The participants tended to overestimate the test distance in both tasks. In DADR, the effect of the adapting distance on the normalized reproduced distance was significant (F(2, 10.0)  4.45, p  =  .041). In addition, as in Experiment 1, there was a significant linear trend (t(10.0)  −2.69, p  =  .022). However, post-hoc pairwise comparisons failed to detect significant difference in the normalized reproduced distance between conditions. These results partially replicated the results of Experiment 1, although underestimation was not evident when the adapting distance was longer than the test distance. In contrast, in TADR, the effect of adapting time on the normalized reproduced distance was not significant (F(2, 10.1)  0.50, p  =  .617), and there was no significant linear trend (t(9.9)  0.19, p  =  .852). The interaction between speed change and the adapting condition was not significant in either DADR or TADR, suggesting that the effect of task-irrelevant temporal information on the reproduced distance did not differ across conditions in either task.

These results seem to suggest that the distance aftereffect only occurs when the distance of the adapting stimuli is fixed and that the aftereffect indeed reflects adaptation to distance rather than time. However, further LMM analysis with fixed-effect factors task (two levels: DADR or TADR) and adapting condition (three levels) indicated no significant interaction between task and adapting condition (F(2, 10.8)  1.82, p  =  .207). Therefore, although the distance aftereffect as in DADR in Experiment 1 was partially replicated, no conclusive evidence was obtained to support the distinctiveness in the effects of distance and time adaptation on distance reproduction.

### Discussion

In Experiment 2, we only measured distance reproduction by examining the DADR and TADR conditions. We did not measure time reproduction, because the purpose of the present study was to examine whether the negative aftereffects can be observed both in the distance and time estimation in a similar manner, and we did not observe the typical pattern of the aftereffect in time estimation in Experiment 1.

Unlike Experiment 1, the effect of task-irrelevant temporal information on reproduced distance was not significant. Again, these results are consistent with previous studies showing that time has little effect on distance estimation (Riemer et al., 2018). In addition, the effect was comparable for DADR and TADR, suggesting that this effect may not explain the difference in the reproduced distance between the two tasks.

The results of DADR suggested that the distance aftereffect was similar to that of Experiment 1. However, while we observed a tendency for overestimation, underestimation was not evident. One possible explanation might be that the reproduction task is sensitive to motor noise, which could have weakened the effect (Petzschner et al., 2015; Shima et al., 2016). It is also possible that the stimulus intensity was not optimal for producing a large aftereffect, because the stimulus parameters in this experiment were largely exploratory. Nevertheless, there was a significant linear trend, which suggests that we replicated the distance aftereffect, albeit with a weaker effect.

On the other hand, the results of TADR suggested that there was no significant aftereffect when the duration of the adapting stimuli was fixed and the distance was varied. This is consistent with our prediction that the aftereffect would be smaller or not present in TADR if the distance aftereffect reflects selective adaptation to the travel distance. However, since the lack of statistical significance does not mean the absence of an effect, the results must be interpreted with caution.

The 2 (DADR or TADR)  3 (three adapting conditions) LMM analysis did not provide evidence to support the distinctiveness in the effects of distance and time adaptation on distance reproduction. One possible explanation for this lack of significance might be that, even though we determined the sample size by the power analysis using the results of experiment 1, the effect size of the interaction was small, leading the sample size to be insufficient to detect the existing effect. Since the sample size for Experiment 2 was determined so that it would be sufficient to replicate the distance aftereffect of DADR in Experiment 1, it could have been underpowered to detect the interaction. Another possible explanation is that the effects of distance and time adaptation on distance reproduction may be indistinguishable in the present experiment. Because the adapting distance/time and travel time/distance are inevitably correlated across conditions in the current experimental manipulation, it is impossible to completely dissociate them. Therefore, future research should examine the nature of distance and time adaptation effects using an experimental paradigm in which distance and time can be manipulated independently.

## General Discussion

In a set of two experiments, we investigated the differences and similarities of the estimation schemes for travel distance and travel time by evaluating the adaptation effect. In Experiment 1, we examined the distance and time adaptation effects using a distance/time reproduction task. A negative aftereffect was observed in the distance reproduction task, while no such effect was observed in the time reproduction task. In Experiment 2, we conducted a cross-dimension adaptation experiment to check whether the negative aftereffect in distance estimation reflected adaptation to distance. The results suggested that the distance aftereffect was significant when the distance of the adapting stimuli was fixed, while the effect was not significant when the time of the adapting stimuli was fixed.

### Repetitive exposure to self-motion has different effects on the distance and time estimates

It has been pointed out that the magnitude estimation of travel distance shows characteristic biases, such as regression and range effects (Petzschner & Glasauer, 2011; Robinson & Wiener, 2021; Thurley & Schild, 2018). These biases are also observed in other spatial, temporal, and numerical magnitudes (Anobile et al., 2012; Anobile et al., 2019; Jazayeri & Shadlen, 2010; Jürgens & Becker, 2011), suggesting a common processing mechanism across different modalities (Petzschner et al., 2015). In addition, many of these magnitudes are known to produce negative aftereffects after prolonged presentation of stimuli. For example, after the repetitive presentation of stimuli with a constant duration, there is a negative aftereffect on duration perception (Heron et al., 2012; Li et al., 2017; Shima et al., 2016). Similar aftereffects have also been reported in the perception of spatial (Dickinson et al., 2017; Pooresmaeili et al., 2013) and numerical magnitudes (Anobile et al., 2018; Burr & Ross, 2008). Here, we report for the first time that a negative aftereffect also occurs in the estimation of the travel distance of one's self-motion. This may provide further evidence in support of the common processing mechanisms for the estimation of different magnitudes.

In contrast, in the estimation of travel time, there was an overestimation after exposure to shorter durations, whereas there was no underestimation after exposure to longer durations, but rather an overestimation, with the overall data showing a quadratic trend. Given that the stimulus parameters in the present experiment were similar to those in our previous study (Shima et al., 2016), which reported a duration aftereffect, this result appears to contradict it. However, unlike the previous study, the stimuli used in the current experiment were complex, involving distance and speed information. Hence, we believe that a simple comparison cannot be made.

Although it is speculative, a possible reason for the quadratic trend could be that it is confounding that we used stimuli that spanned sub-second (i.e., shorter than 1 s) and supra-second (i.e., longer than 1 s) as the adapting stimuli, and that we did not dissociate the adaptation effect from serial dependence. It has been suggested that there are distinct time-processing systems divided at a boundary of approximately 1 s (Buhusi & Meck, 2005; Hayashi et al., 2014; Wiener et al., 2011). Although our previous study suggested that duration aftereffect occurs across sub- and supra-second stimuli (Shima et al., 2016), other previous studies have reported duration aftereffects mainly in sub-seconds, and the mechanism of duration aftereffect across sub- and supra-second stimuli remains unclear.

It is also known that the preceding stimulus has a bidirectional effect on the estimation of the current stimulus, namely, negative aftereffects repel perception away from previous stimuli, while positive serial dependence attracts decisions toward previous stimuli (Fritsche et al., 2017). Thus, in the present experiment, the quadratic trend could be explained if we consider that the negative aftereffect dominates in the condition with shorter adapting stimuli (where the adapting stimuli were sub-second) and the positive serial dependence dominated in the condition with longer adapting stimuli (where the adapting stimuli were supra-second). However, since the current experiment does not allow us to distinguish negative aftereffects from positive serial dependence, we cannot infer what the observed effects originate from. Future research is needed to dissociate these two estimation biases. Nevertheless, the different biases in the distance and time estimates indicate that repetitive exposure to self-motion has different effects on the estimation of spatial and temporal aspects of the current movement, suggesting that they may be processed by partially separable systems.

### The distance aftereffect does not originate from time adaptation alone

In Experiment 2, the distance aftereffect was replicated when the distance of the adapting stimuli was fixed, but not when the distance of the adapting stimuli was varied. However, we did not find an interaction between the effects of the two tasks. While these results are somewhat confusing and inconclusive, they do suggest that the effect cannot be explained by adaptation to time alone. If the aftereffect in the distance reproduction is caused as a result of adaptation to the duration of the stimuli, one would expect a large effect when the duration of the adapting stimuli is fixed compared with when the distance of the adapting stimuli is fixed. However, this was not the case in the present study. While the relationship between the observed effect and its neural mechanism remains speculative, one possible interpretation of this result is that the distance aftereffect may be caused by an adaptation of the system that processes spatial information separately from temporal information. For example, electrophysiological studies in monkeys have found neurons in the frontal and parietal cortices that tune to specific distances and lengths in spatial discrimination tasks (Genovesio et al., 2011; Marcos et al., 2016; Tudusciuc & Nieder, 2007; Tudusciuc & Nieder, 2009). In the context of spatial navigation, Kraus et al., reported that, in rodents, some neurons in the hippocampus and grid cells in the entorhinal cortex exhibit firing patterns that are tuned to distance travelled independently of time elapsed while rats are running on a treadmill (Kraus et al., 2013; Kraus et al., 2015).

On the other hand, it should be noted that the negative aftereffect may not reflect perceptual bias owing to sensory adaptation of neurons that encode a particular stimulus property, but may be related to higher-order processing such as memory and decision biases (Postman & Miller, 1945; Storrs, 2015). In particular, owing to the nature of the adaptation paradigm, we employed the reproduction task, which has been widely used to evaluate distance estimates (Frenz & Lappe, 2005; Mossio et al., 2008; Petzschner & Glasauer, 2011; Riemer et al., 2018; Robinson & Wiener, 2021; Thurley & Schild, 2018; Wiener et al., 2016). However, it has been pointed out that the aftereffects observed with this method are likely to reflect cognitive bias rather than perceptual bias (Storrs, 2015). This is because, if the aftereffect truly reflects the appearance of the stimulus, then the reference stimulus in the reproduction phase may also be affected by perceptual bias; thus, the effect may be cancelled out (Firestone & Scholl, 2014). Given that negative aftereffects in many other magnitudes have been suggested to have a “channel-based” mechanism as their neural basis and are likely to reflect perceptual rather than cognitive aftereffects (Castaldi et al., 2016; Hayashi & Ivry, 2020; Pooresmaeili et al., 2013), they might have a different origin from the aftereffects reported here. Future research is necessary to address this critical question.

### Asymmetry between the estimation of distance and time

Throughout the experiment, the results of the correlation analysis between the speed change and reproduced distance/time suggested that the space–time interference effect was asymmetric between the distance and time tasks. Time estimation was affected by distance, whereas distance estimation was not affected by time, or if it was, the effect was smaller than the reverse. This finding is consistent with previous studies suggesting the superiority of the processing of spatial information over the processing of temporal information in magnitude estimation (Casasanto et al., 2010; Casasanto & Boroditsky, 2008; Riemer et al., 2018). The asymmetry was also evident in the precision of the estimation, showing that the distance reproduction was less precise than the time reproduction. These asymmetries might be due to the difference in the estimation strategy between the distance and time tasks. While time reproduction can be performed in a pre-planned manner (Jazayeri & Shadlen, 2015; Robinson & Wiener, 2021), distance reproduction needs to take speed into account, and the estimate may be made based on evidence accumulated from an optic flow rather than in a pre-planned manner (Mossio et al., 2008; Robinson & Wiener, 2021; Zajac et al., 2019). For example, in the distance task, the negative correlation between the speed change and the reproduced distance (i.e., participants reproduced a shorter distance when the speed of the reproduction phase was faster than that of the test phase) might be due to the strategy of “stopping button pressing early as the speed became faster.” This possible difference in the estimation strategy could lead to a difference in the observed adaptation effect between the two tasks. In the present study, this interference effect was taken into account by statistical analysis within each task, but it would be interesting to investigate how the adaptation effect changes when pre-planned reproduction is made possible in the distance task as well, for example, by placing landmarks in the distance estimation task (Brown et al., 2016).

It remains to be tested whether and how the adaptation effect develops with different stimulus parameters, that is, adapting distance/time, test distance/time, and range of speed. It should be noted that underestimation was not evident in the DADR in Experiment 2. Future studies should parametrically examine the stimulus parameters that produce the strongest aftereffect and explore a less noisy method than reproduction.

Finally, although the distance estimation in the VR environment is considered to be mainly based on optic flow (Mossio et al., 2008; Warren et al., 2001; Zajac et al., 2019), other idiothetic cues (e.g., proprioceptive input) are also important when estimating the distance in actual path integration (Chrastil & Warren, 2014; Taube et al., 2013). Therefore, further research is needed to elucidate whether the present results can be extended to real-world environments.

### Conclusions

We observed a negative aftereffect in the estimation of the distance travelled by one's self-motion. The estimation of elapsed time exhibited different trends: The estimation of elapsed time after adaptation showed a mixed outcome; one resembled a negative aftereffect, and one resembled a positive serial dependence. Our results highlight the similarity between the estimation of distance and other magnitude dimensions, and support the idea that spatial and temporal processing relies on partially separable mechanisms.

## Supplemental Material

sj-docx-1-ipe-10.1177_20416695221078878 - Supplemental material for Partially Separable Aspects of Spatial and Temporal Estimations in Virtual Navigation as Revealed by Adaptation Click here for additional data file. Supplemental material, sj-docx-1-ipe-10.1177_20416695221078878 for Partially Separable Aspects of Spatial and Temporal Estimations in Virtual Navigation as Revealed by Adaptation by Taku Otsuka and Yuko Yotsumoto in i-Perception
