# Supplementary material for: Partially Separable Aspects of Spatial and Temporal Estimations in Virtual Navigation as Revealed by Adaptation
Source: Iperception. 2022 Feb 24;13(1):20416695221078878. doi: 10.1177/20416695221078878 (PMC8883378; doi:10.1177/20416695221078878)
Supplement: sj-docx-1-ipe-10.1177_20416695221078878 - Supplemental material for Partially Separable Aspects of Spatial and Temporal Estimations in Virtual Navigation as Revealed by Adaptation [file sj-docx-1-ipe-10.1177_20416695221078878.docx]

**Supplementary Materials**

**Effects of task-irrelevant dimension on the response**

**
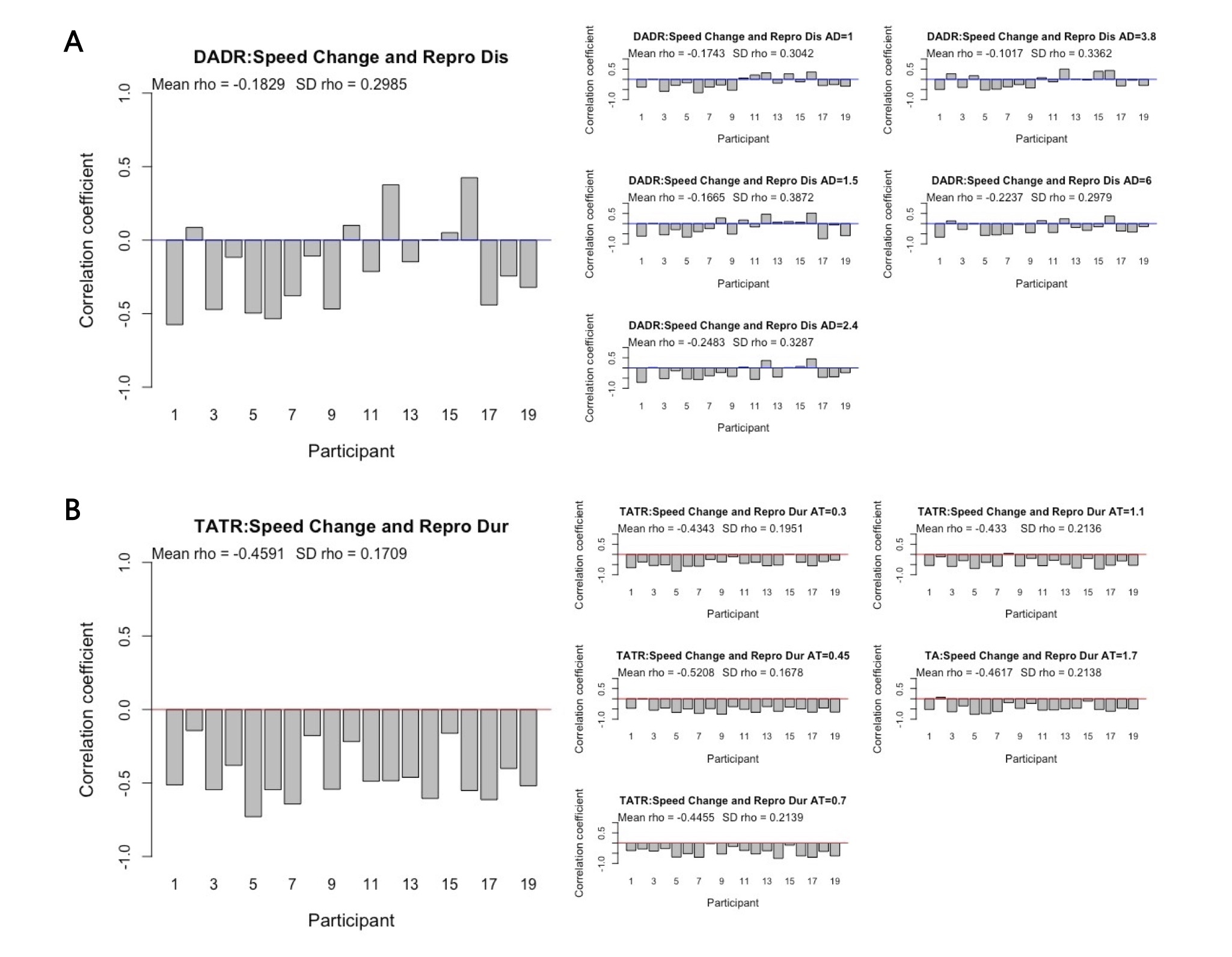

Supplementary Figure 1.** Effects of task-irrelevant dimension on the response in Experiment 1. A, B: Spearman correlation coefficients between the speed change (reproduction phase minus test phase) and the reproduced distance and time in the DADR and TATR, respectively. A non-zero correlation indicates that a task-irrelevant dimension (time/distance) affected the primary task response (distance/time). The left panel shows the across-condition mean of the correlation coefficients, and the right panel shows the correlation for each adapting condition, separately for each participant. DADR = distance adaptation–distance reproduction. TATR = time adaptation–time reproduction.

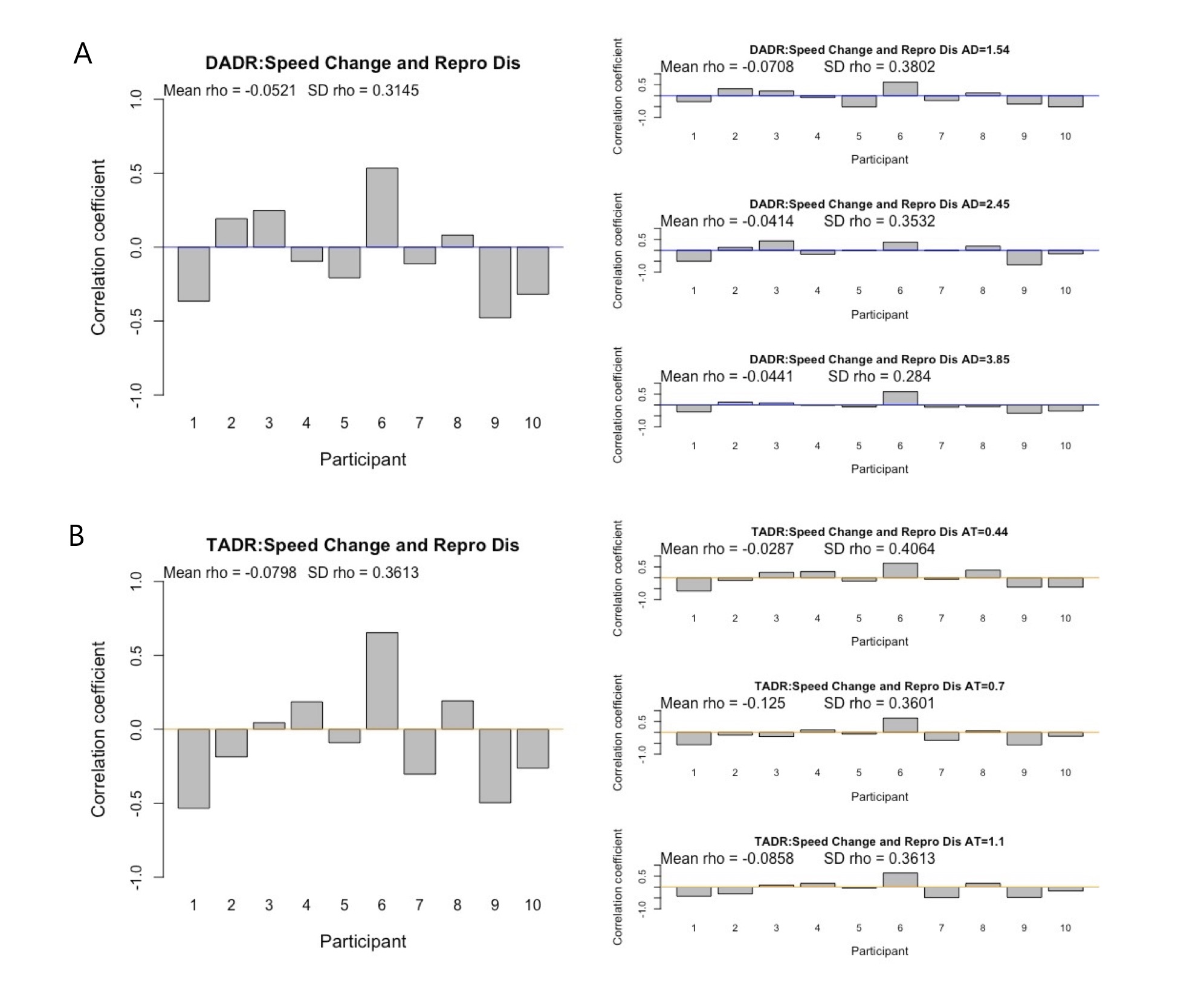

**Supplementary Figure 2.** Effects of task-irrelevant dimension on the response in Experiment 2. A, B: Spearman correlation coefficients between the speed change (reproduction phase minus test phase) and the reproduced distance in the DADR and TADR, respectively. A non-zero correlation indicates that a task-irrelevant dimension (time) affected the primary task response (distance). The left panel shows the across-condition mean of the correlation coefficients, and the right panel shows the correlation for each adapting condition, separately for each participant. DADR = distance adaptation–distance reproduction. TADR = time adaptation–distance reproduction.

**Correlation of the adaptation effects for distance and time, following Anobile et al. (2018)**


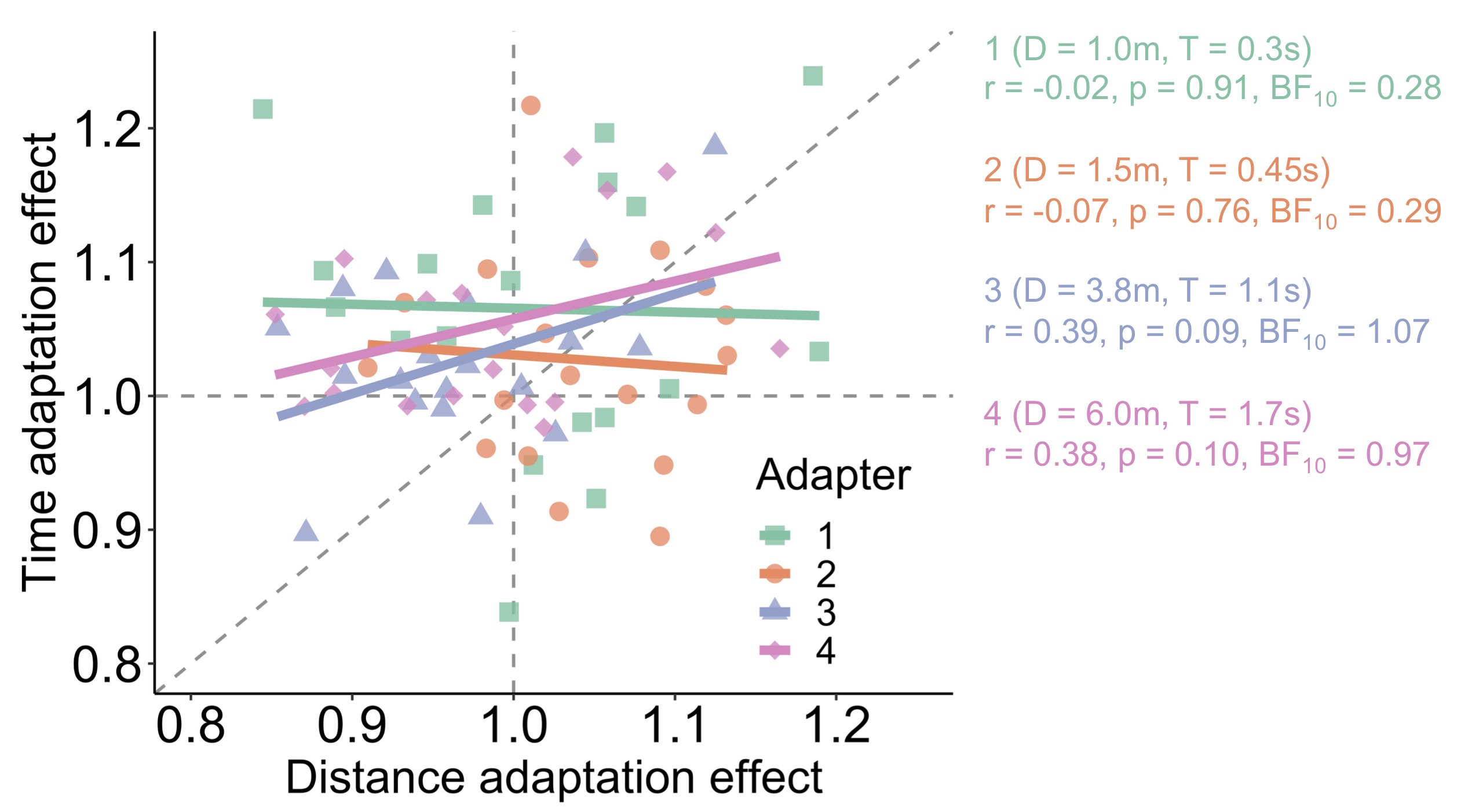

**Supplementary Figure 3.** Pearson correlation between adaptation effects in Experiment1. Distance and time adaptation effects are separated into four adapter levels. Each dot represents the average *normalized reproduced distance/time* for each participant. BF_10_ (Bayes factor) greater than 1 supports a correlation, and BF_10_ less than 1 supports a lack of correlation.

In an additional exploratory analysis, following Anobile et al. (2018), we examined whether the adaptation effects for distance and time in Experiment 1 were correlated across participants. Separately for each participant and each of the four adapting conditions (1…Distance = 1.0 m, Time = 0.3 s; 2…Distance = 1.5 m, Time = 0.45 s; 3…Distance = 3.8 m, Time = 1.1 s; 4…Distance = 6.0 m, Time = 1.7 s), we obtained the mean of *the normalized reproduced distance/time* and calculated the Pearson correlation coefficient. The significance of correlations was calculated by p-values and also by Bayes Factor (Wetzels & Wagenmakers, 2012) using JASP (JASP Team, 2020). The resulting BF_10_ represents how strongly the data support the alternative hypothesis that there is a correlation between the two effects. BF_10_ (Bayes factor) greater than 1 supports the alternative hypothesis, and BF_10_ less than 1 supports the null hypothesis.

Supplementary Figure 3 shows the time against the distance adaptation effects. The correlations were not significant for either condition (p > .05). Bayes factor indicated that there was moderate evidence favoring the null hypothesis in the conditions where the adapting stimulus was shorter than the test stimulus (Pearson’s *r* = -0.02, BF_10_ = 0.28; Pearson’s *r* = -0.07, BF_10_ = 0.29), but no strong evidence to support either hypothesis in the conditions where the adapting stimulus was longer than the test stimulus (Pearson’s *r* = 0.39, BF_10_ = 1.07; Pearson’s *r* = 0.38, BF_10_ = 0.97).

Taken together, the results of the null hypothesis test and the Bayes factor did not provide conclusive evidence either in support of or against a correlation between the adaptation effects for distance and time.
